# Facile Preparation of Carbon Nanotube-Cu_2_O Nanocomposites as New Catalyst Materials for Reduction of P-Nitrophenol

**DOI:** 10.1186/s11671-019-2914-1

**Published:** 2019-03-05

**Authors:** Yao Feng, Tifeng Jiao, Juanjuan Yin, Lun Zhang, Lexin Zhang, Jingxin Zhou, Qiuming Peng

**Affiliations:** 1 State Key Laboratory of Metastable Materials Science and Technology, Yanshan University, Qinhuangdao, 066004 People’s Republic of China; 2 Hebei Key Laboratory of Applied Chemistry, School of Environmental and Chemical Engineering, Yanshan University, Qinhuangdao, 066004 People’s Republic of China

**Keywords:** Nanocomposite, Cuprous oxide, Carbon nanotube, Catalytic reduction, P-nitrophenol

## Abstract

The effective synthesis and self-assembly of nanocomposites were of key importance for a broad range of nanomaterial applications. In this work, new carbon nanotube (CNT)-Cu_2_O nanocomposites were successfully synthesized via a facile approach. CNT was selected as the anchoring substrate to load Cu_2_O nanoparticles to prepare composite catalysts with well stability and good reusability. It is discovered that the prepared CNT-Cu_2_O nanocomposite materials could be effectively controlled via regulating preparation temperature and time without the use of any stabilizing agents. The nanostructures of synthesized composites were well characterized by many techniques, such as scanning electron microscopy (SEM), transmission electron microscopy (TEM), and X-ray diffraction (XRD). And the prepared CNT-Cu_2_O nanocomposites with optimized preparation conditions as new catalyst displayed excellent catalytic performance on the reduction reaction of p-nitrophenol, demonstrating potential applications for environmental governance and composite materials.

## Background

Since the discovery of carbon nanotubes (CNT), the related research and applications for catalysts [1, 2], flexible supercapacitors [3], electronic sensors [4], and sustainable wastewater treatment [5] have been widely explored. It is well known that CNTs are special materials owing to high chemical stability, well electrical conductivity, large specific surface area, and extremely high mechanical strength [6]. These special properties make CNTs a great favor to researchers. In recent years, more research work on CNT catalysis have been conducted, most of which are related to composites with transition metals. For example, Karimi-Maleh et al. have synthesized CuO/CNTs nanocomposite by chemical precipitation method used as high adhesive carbon paste electrode [7, 8]. With the recent development of nanoscience and nanotechnology, developing a facile and low-cost strategy for synthesizing multi-functional carbon material is an important challenge. At the same time, more and more nanomaterials have been researched for improving catalytic properties, such as Pd [9], TiO [10], Mo [11], Zn [12], Au [13], and Ag [14]. Liu et al. used zinc to fabricate chloroplast mimics by the self-assembled approach, which was beneficial for photoenzymatic reaction in sustainable fuel synthesis [15]. Silver nanoparticles, for example, are now widely used as catalysts due to high reactivity and selectivity [16, 17]. In addition, Pt nanoparticles could serve as an electron separator [18]. Pt and TiO_2_ acted as self-mineralization to reform the fiber bundles architecture [19]. Liu et al. researched the peptide-porphyrin co-assemblies; in this, they presented Pt nanoparticles easily mineralized [20]. A dendritic pyrenyl-moiety-decorated hyperbranched polyglycidol (pHBP) was prepared by Li and coworkers, which accomplished to functionalize CNTs via a non-covalent (non-destructive) process, after that Au, Ag, and Pt nanoparticles with uniform SiO_2_, GeO_2_, and TiO_2_ coatings were deposited in situ onto the as-prepared CNT/pHBP hybrids [21]. Novel CNT/pHBP/Au hybrids and CNT/pHBP/Pt had been reported and showed excellent catalytic activity for 4-NP reduction [13, 21]. In addition, the research group of Szekely achieved an excellent work about azido-derivatized cinchona-squaramide bifunctional catalyst grafted to the surface of polybenzimidazole-based nanofiltration membrane, which confirmed the change in geometry and increased secondary interactions, and enhanced the catalytic effect [22].

On the other hand, copper nanocrystals belonged to low-cost and higher abundance materials used as catalysts. The size and shape impacted on the catalytic activity, while the surface microstructures and arrangement of Cu atoms on the surface also determined the catalytic results [23]. Meldal [24] and Sharpless [25] had explored the ability of Cu^+^ salts at room temperature or with moderate heating to speed up some cycloaddition reaction [26]. With functionalization of Cu catalysts, Cu nanomaterials were effectively used for electrocatalysis, photocatalysis, and CO_2_ catalysis. For example, previous reports put forward that Cu was applied to the field of visible light active photocatalyst [27]. In that work, the simultaneous functionalization of Cu and Cd catalysts and photocatalytic CO_2_ reduction were realized successfully. Copper nanostructures were also used in catalytic oxidation reactions, but these mechanisms are different from other metal catalysts [28]. Because CNTs demonstrated high mechanical strength, high thermal and electrical conductivity and adsorption, unique nanostructure, mechanical and thermal properties, and hydrophobicity, many researchers applied CNTs as templates to support for heterogeneous catalysts [29]. Hybrid nanoflower composites mixed with CNTs showed high enzyme wiring efficiency and electron transfer rate which could be used in the field of fabricating enzymatic biofuel cells [30]. In addition, Esumi’s group explored the dendrimer-encapsulated Au NPs for 4-NP reduction, but the results showed the process influenced by the concentration and generation of the dendrimers [13, 31–33]. At the same time, some research work about CNT and Cu composites had been reported. Typically, Leggiero et al. found that the seeded Cu using the CVD method and electrodeposited with CNT achieved excellent conductors [34]. Cho et al. explored CNT and metal matrix composite-compounded chromium carbide by in situ formation, which achieved incompatible properties including electrical conductivity and temperature coefficient of resistance [35].

Herein, we report the synthesis of stable CNT-Cu_2_O nanocomposites by a simple and easy way of preparation. Cu_2_O nanocrystals were prepared from CuCl precursor. We adjusted different preparation temperatures and time to regulate the sizes of the formed Cu nanostructures. The method introduced in the previous report is relatively complexed, while the preparation approach in the present case seemed simple and eco-friendly with low material cost. Also, the as-prepared composite materials could be used as new catalytic materials and utilized for the reduction reaction of 4-NP [36]. Particularly, our study could display great potential applications in the wastewater treatment field and composite catalyst materials field.

## Methods

The experiment used materials, multi-walled amination carbon nanotubes (95%, inner diameter of 3–5 nm, external diameter of 8–15 nm, length of 50 μm), cuprous chloride (97%, CuCl), and cupric chloride (98%, CuCl_2_) were purchased from Aladdin Chemicals. Sodium hydroxide (96%, NaOH) was purchased from Tianjin Kermel Chemical Reagent. l-ascorbic acid (99.7%), sodium borohydride (98%, NaBH_4_), and p-nitrophenol (98%, 4-NP) were purchased from Shanghai Hushi Reagent. All used solvents were of analytical grade and directly used without further treatment.

The targeted nanocomposites were synthesized by the following procedure: the mixture of 100 mg carboxylated carbon nanotubes and 100 mg CuCl solid was added into 250 mL deionized water in a clean beaker with magnetic string (300 r/min) for 20 min at 30 °C. Subsequently, keeping stirring for 1 h, 0.88 g ascorbic acid and 5.0 mL NaOH (1 M) were added into the above mixing solution. Then, the solid was washed with 100 mL ethanol and 100 mL deionized water for several times and was collected by drying in a vacuum at 50 °C for 48 h [16]. For comparison, different experimental conditions were controlled at 30 °C with stirring for 6 h or at 60 °C with stirring for 1 h. All the obtained samples were dried in a vacuum at 50 °C for 48 h.

The catalytic experiments were carried out and detected according to the previous reports [16]. In catalytic experiments, 0.0174 g 4-NP and 0.1892 g NaBH_4_ were dissolved into 25 mL deionized water, respectively, and about 16 mL of 4-nitrophenol aqueous solution (0.313 mM) and a freshly prepared 15 mL sodium borohydride solution were added into a beaker at room temperature [36, 37]. Then, the synthesized CNT/Cu_2_O composites (10 mg) were dispersed in the above-prepared solution to obtain a suspension. For standardizing instrument, 1.5 mL deionized water was added into a quartz cuvette and monitored using a UV-vis spectrophotometer at a wavelength from 220 to 550 nm. After that, every 4 min intervals, 1.5 mL supernatant liquid was monitored and recycled, which kept constant concentration in the reaction system. After the catalytic reaction, the used catalyst was recovered by centrifugation and washed with ethanol and water several times.

The morphologies of prepared composites were analyzed by employing a field-emission scanning electron microscope (SEM) (S-4800II, Hitachi, Japan) with the accelerating voltage of 15 kV. EDXS analysis was typically performed at 200 kV acceleration using an Oxford Link-ISIS X-ray EDXS microanalysis system attached to SEM. Transmission electron microscopy (TEM, HT7700, Hitachi High-Technologies Corporation) was investigated with commercial 300-mesh copper grids. With an accelerating voltage of 200 kV, elemental mappings in composites were distinguished utilized to X-ray spectroscopy (EDXS). The composites were performed with a motorized sample stage of Horiba Jobin Yvon Xplora PLUS confocal Raman microscope equipped [38–42]. X-ray diffraction (XRD) analysis was investigated on an X-ray diffractometer equipped with a Cu Kα X-ray radiation source and a Bragg diffraction setup (SmartLab, Rigaku, Japan).

## Results and Discussion

**Fig. 1:**
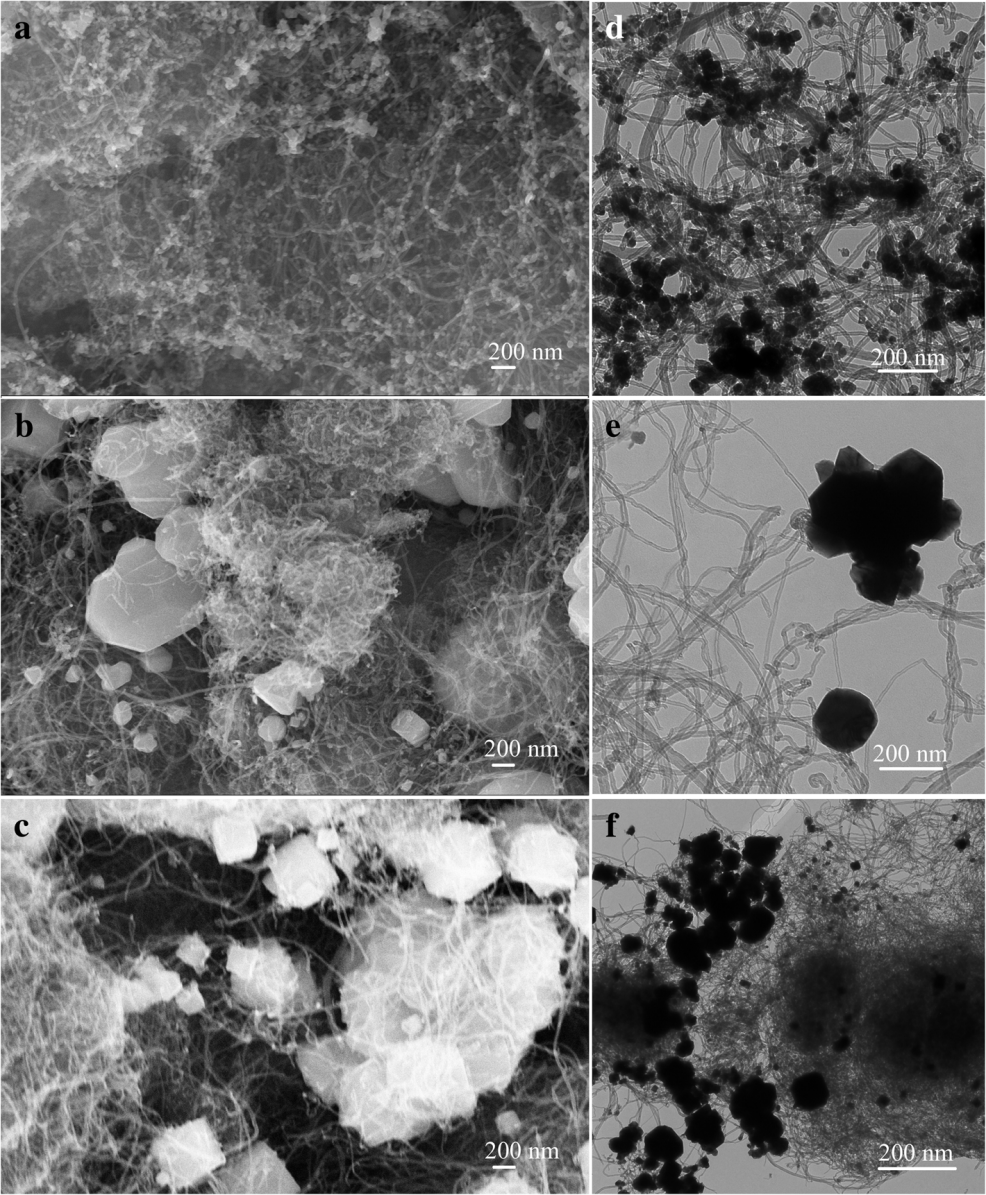
SEM and TEM images of the synthesized CNT-Cu_2_O nanocomposites. **a**, **d** CNT-Cu-30-1. **b**, **e** CNT-Cu-60-1. **c**, **f** CNT-Cu-30-6

**Fig. 2:**
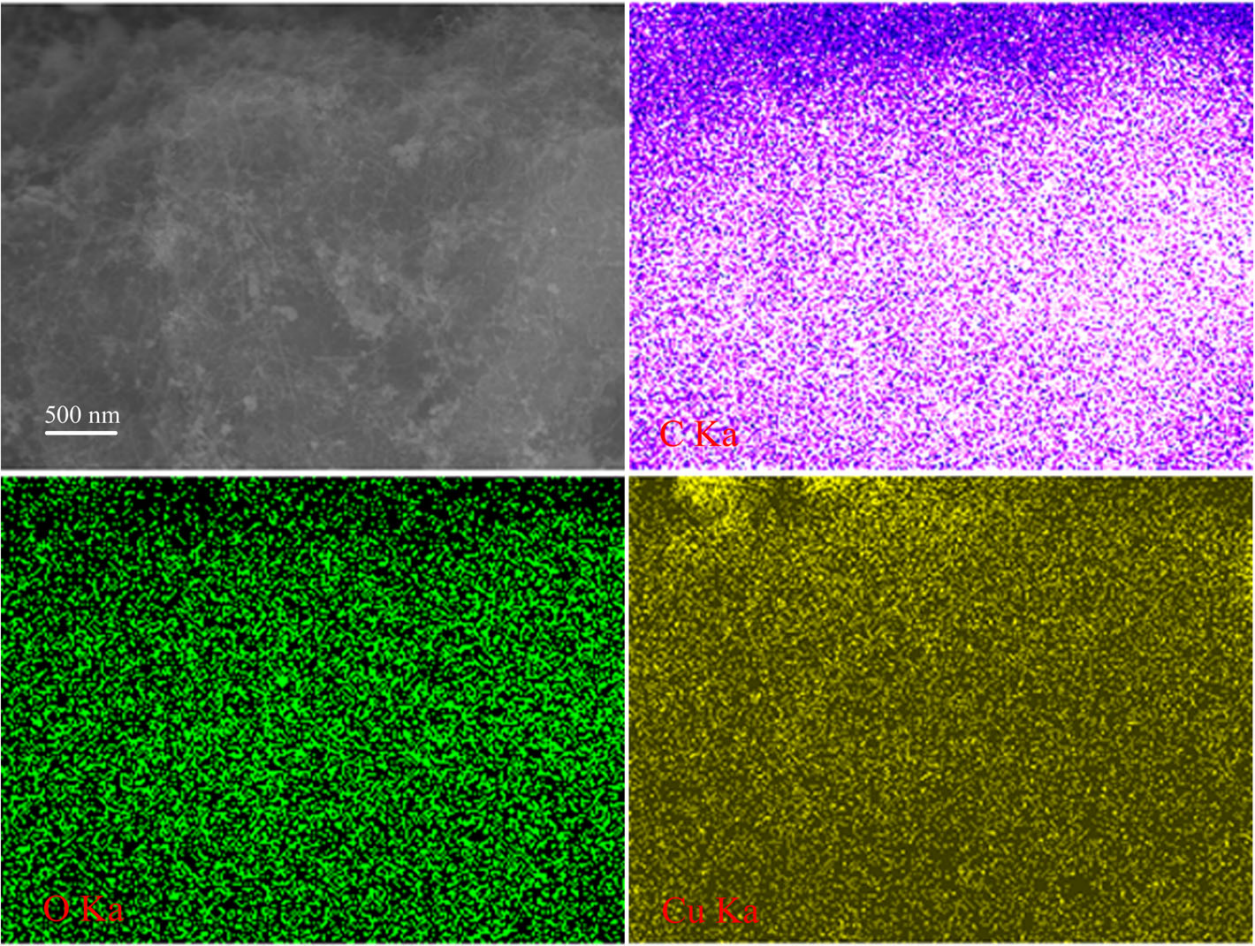
SEM image of CNT-Cu-30-1 nanocomposite and elemental mappings of C, O, and Cu

Firstly, Fig. 1 demonstrated the nanostructures of the synthesized CNT-Cu_2_O nanocomposites via carbon nanotubes and cuprous chloride. After attempting the characterization of different parameters, the optimal products were obtained under different reaction factors. As shown in Fig. 1a, d, with reaction temperature 30 °C and stirring for 1 h, the size of Cu_2_O nanocrystallines showed about 30–50 nm evenly distributed on the surface of nanotubes in the synthesized CNT-Cu-30-1 composite. In addition, the obtained composites under 30 °C and stirred for 6 h or 60 °C for 1 h, named as CNT-Cu-60-1 and CNT-Cu-30-6, have also been investigated, displaying big blocks of Cu_2_O nanocrystalline particles even with the diameters at the micrometer scale. In order to further analyze the component dispersion of the prepared CNT-Cu-30-1 composite, we examined the morphologies by using EDS map scanning. Figure 2 showed the SEM image of CNT-Cu-30-1 nanocomposite and elemental mappings of C, O and Cu elements. The obtained results demonstrated the used carbon nanotubes could serve as a good carrier while the formed Cu_2_O nanoparticles evenly adhered on the surface of CNT, which could be speculated to show good catalytic performances.

**Fig. 3:**
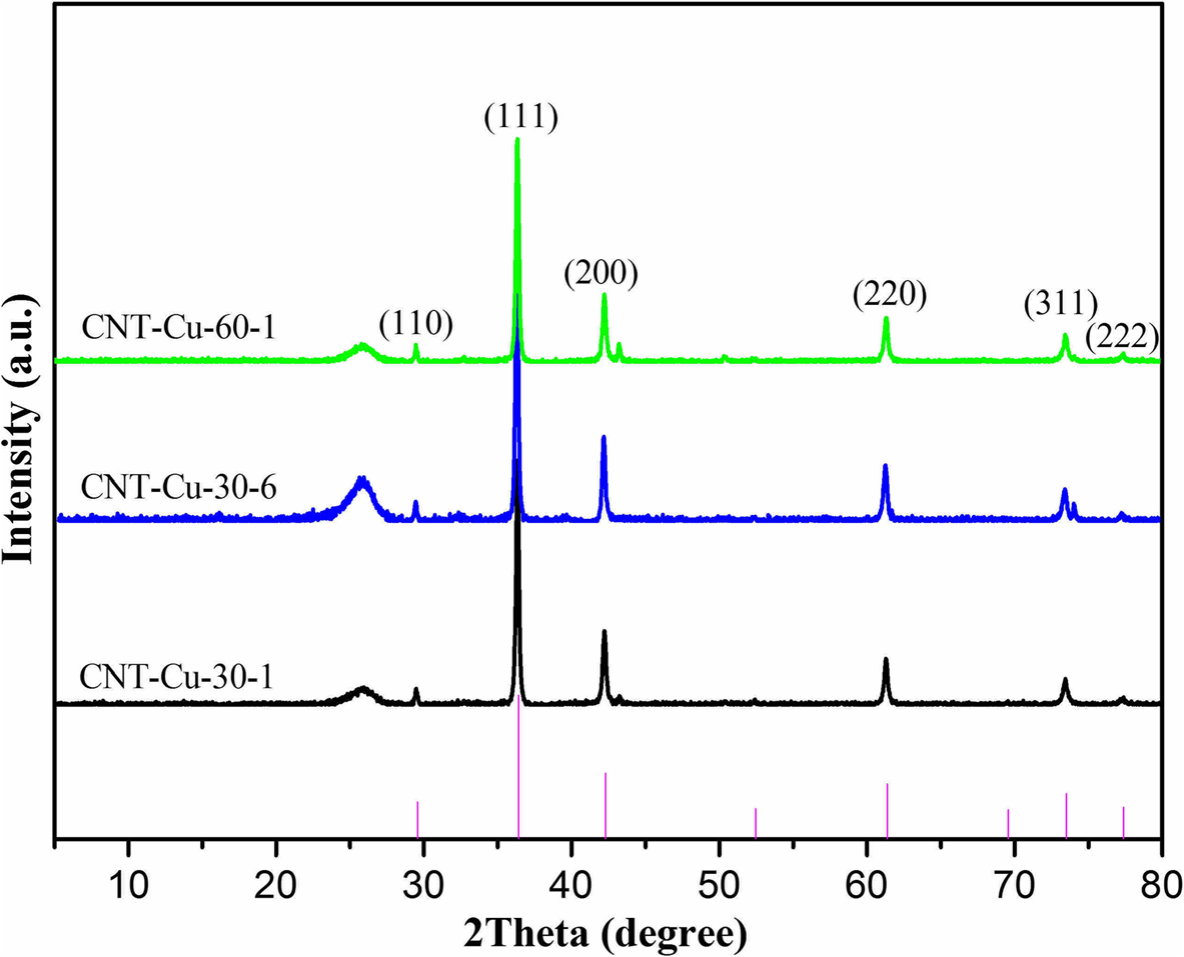
XRD curves of the synthesized CNT-Cu_2_O nanocomposites

**Fig. 4:**
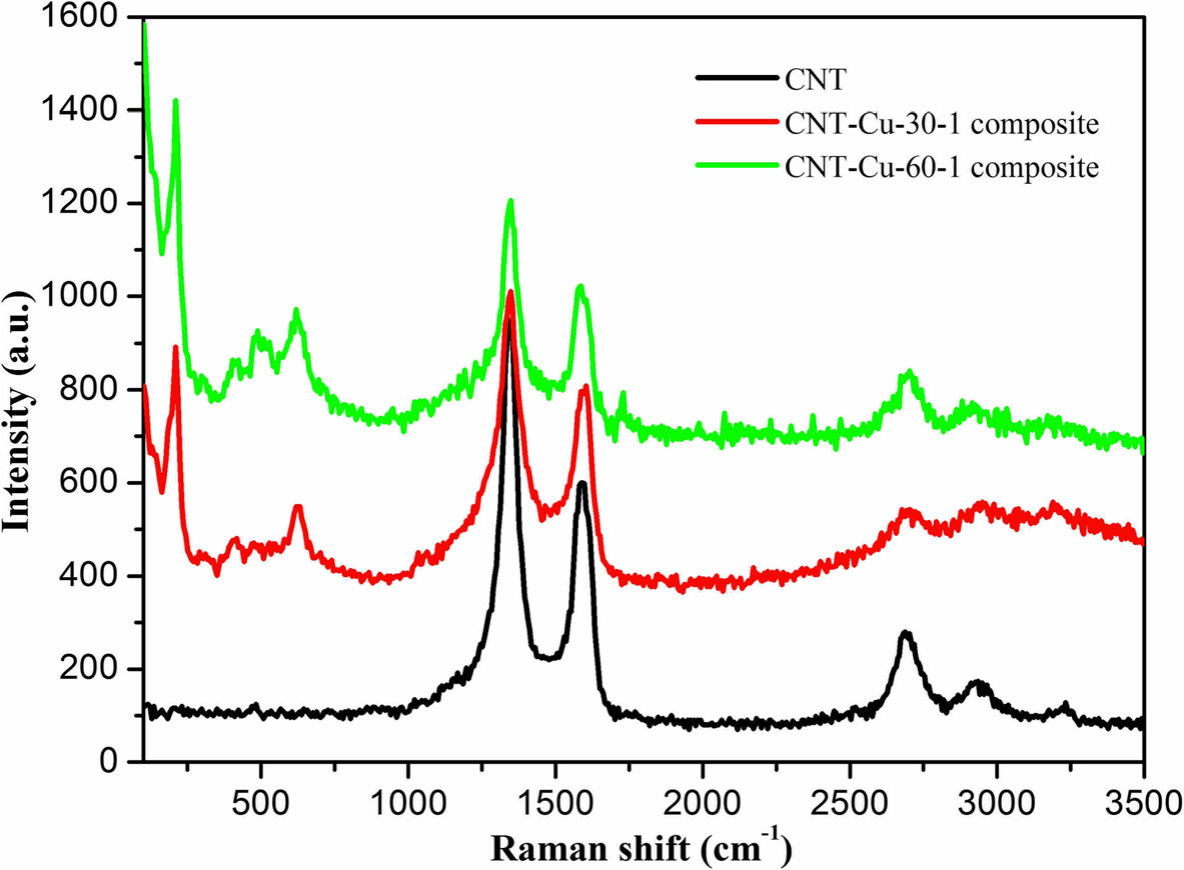
Raman spectra of the CNT and synthesized CNT-Cu_2_O nanocomposites

Next, the synthesized CNT-Cu_2_O nanocomposites were characterized by XRD technique, as shown in Fig. 3. It is easily observed that the characteristic diffraction peak of CNT appeared at 2*θ* values of 26°, which could be indexed to PDF26–1079 owing to its carbon nanotube structure. In addition, many strong and sharp characteristic peaks could be assigned to Cu_2_O nanocrystalline indexed to PDF05–0667. Apparently, all obtained composite samples showed the same characteristic peaks without any other impurities. It was interesting to note that for exploring the stability of Cu_2_O nanoparticles, the obtained composite after 1 month was analyzed repeatedly by XRD. And the obtained result demonstrated the same curves, indicating the well stability of synthesized Cu_2_O nanoparticles. In addition, the Raman spectra of CNT and the synthesized CNT-Cu_2_O nanocomposites were investigated and shown in Fig. 4. Comparing CNT spectra with CNT-Cu-30-1 and CNT-Cu-60-1 spectra, all curves showed two distinct visible peaks (G and D) and a smaller one (2D), proving the existence of a carbon-based matrix. D peak (1344 cm^−1^) represented the defects and disorders in CNT, and G peak (1605 cm^−1^) indicated the result of disorder in sp^2^-hybridized carbon systems. Moreover, 2D peak (2693 cm^−1^) could be attributed to two-phonon lattice vibrations in CNT structure. It should be noted that in Fig. 4, the intensity ratio of the D and G peaks (*I*_*D*_/*I*_*G*_) for CNT showed the value of 1.64. However, the value of *I*_*D*_/*I*_*G*_ of CNT-Cu-30-1 composite (1.34) suggested to be smaller than CNT but larger than CNT-Cu-60-1 composite (1.29). Owing to the higher the ratio, the bigger the defect of carbon crystal, which further demonstrated that the crystallinity of CNT-Cu-60-1 composite seemed larger than CNT-Cu-30-1, matching well with the results from the SEM characterization. On the other hand, characteristic peaks at 223 cm^−1^ and 485 cm^−1^ represented lattice vibrations in Cu_2_O crystal.

**Fig. 5:**
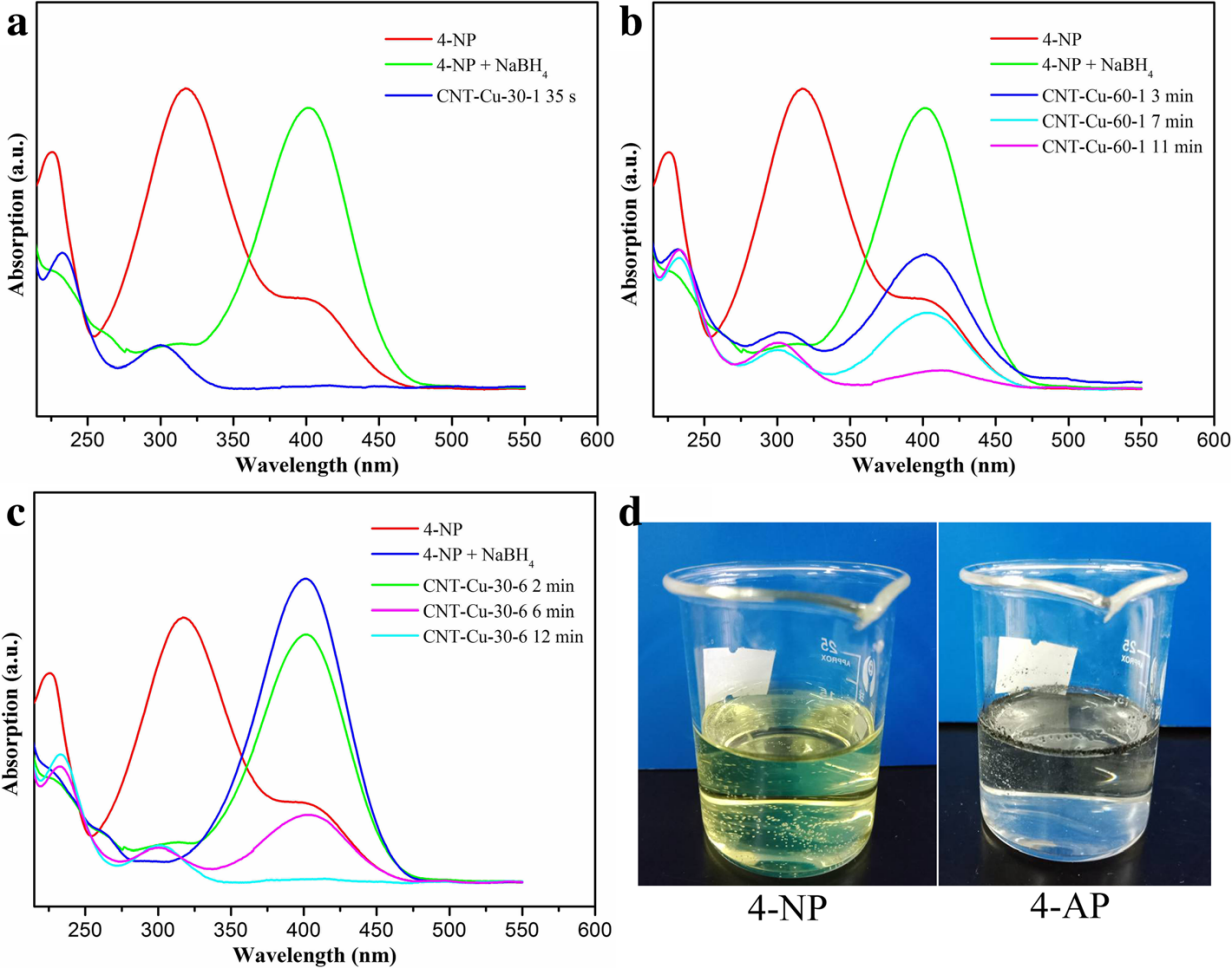
Catalytic reduction of 4-NP via present synthesized CNT-Cu_2_O nanocomposites. **a** CNT-Cu-30-1. **b** CNT-Cu-60-1. **c** CNT-Cu-30-6. **d** The photos of 4-NP solution before and after the catalytic process

The obtained CNT-Cu_2_O nanocomposites have been utilized to reduce 4-nitrophenolate (4-NP) to 4-aminophenol (4-AP) solution in the presence of NaBH_4_ as a typical reaction model. It was well reported that UV-vis spectroscopy was a distinct method to monitor 4-NP reduction reaction [23, 36, 38], which was widely studied. At first, the 4-NP solution with fresh aqueous NaBH_4_ was monitored by UV-vis spectroscopy, as shown in Fig. 5. The maximum absorption peak of 4-NP was located at 314 nm. After the addition of NaBH_4_ solution without the catalyst, the solution appeared bright yellow with peak position transferred to 401 nm, indicating the formation of 4-nitrophenolate [36]. As shown in Fig. 5a, the prepared CNT-Cu-30-1 composite could completely catalyze 4-NP mixtures with NaBH_4_ to produce 4-aminophenol within 35 s, which could be mainly owing to the high catalytic capability of Cu_2_O nanoparticles in this composite. In addition, when the catalysts of CNT-Cu-60-1 or CNT-Cu-30-6 were added into the reaction mixture, the maximum absorbance peak at 401 gradually disappeared after about 11–12 min, which suggested the formation of product 4-AP. At the same time, as presented in Fig. 5d, the color of the 4-NP solution with NaBH_4_ changed from bright yellow to colorless state after the catalytic process, indicating the completion of the catalytic process.

**Fig. 6:**
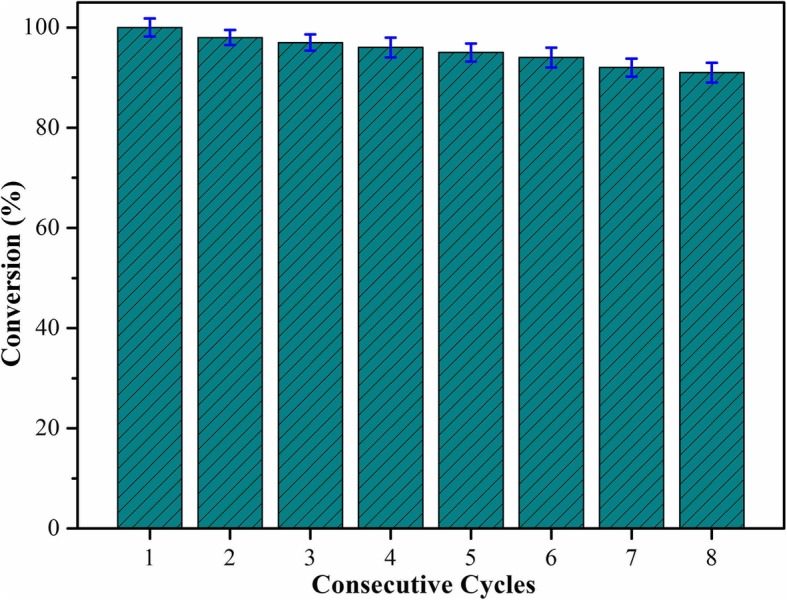
Reusability test of CNT-Cu-30-1 nanocomposite as a catalyst for the reduction of 4-nitrophenol with NaBH_4_

**Fig. 7:**
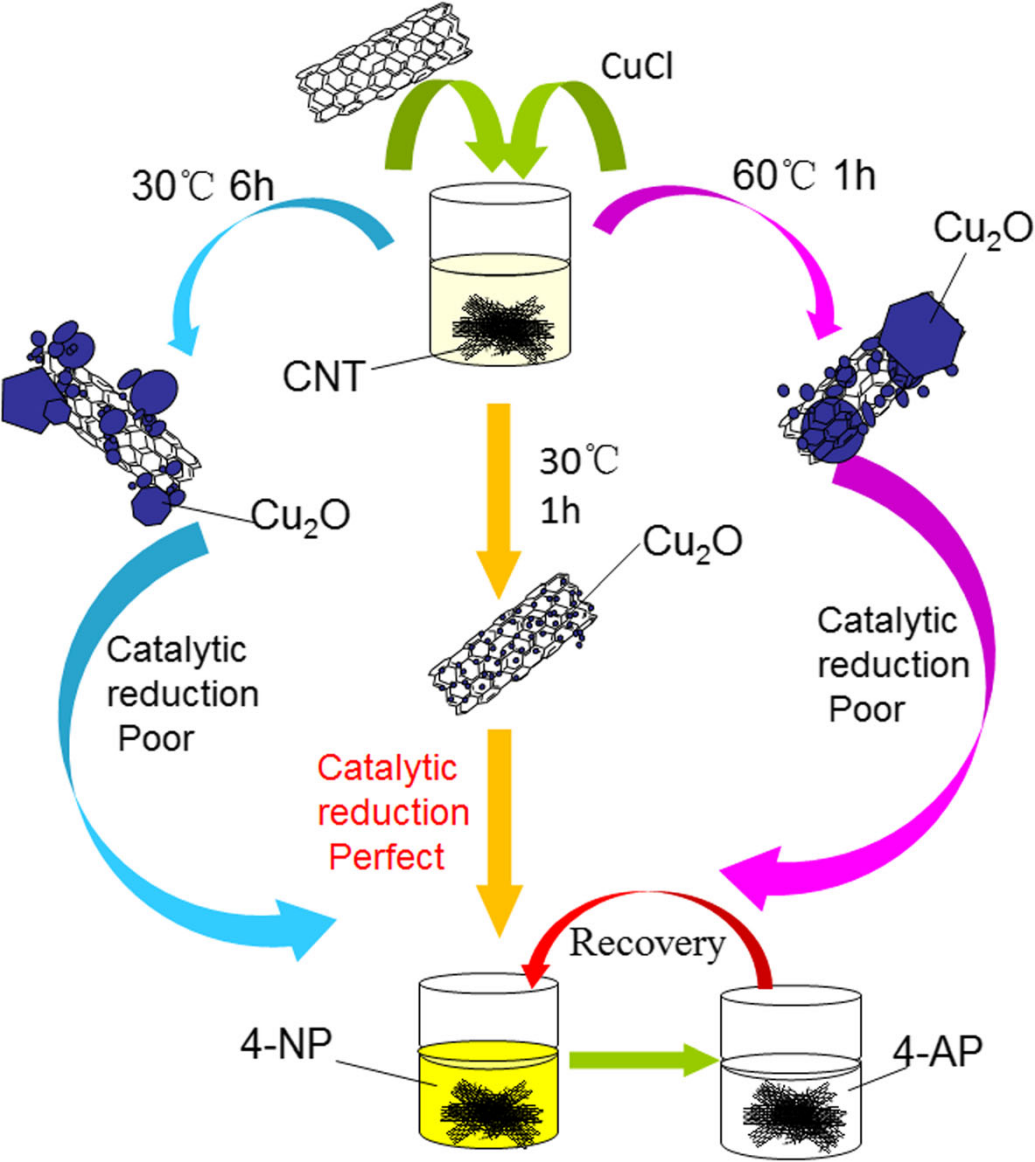
Schematic illustration of the fabrication and catalytic reduction of the synthesized CNT-Cu_2_O nanocomposites

It seemed an important indicator for catalyst materials to possess excellent stability and reutilization performances [43–52]. The continuous process made it sustainable and gained increasing attention, which seemed better than the batch process. Improving the circulation ability of composite helped to reduce the production cost. Thus, based on the above results of catalytic tests, we investigated the reusability of the synthesized CNT-Cu-30-1 nanocomposite as a catalyst for the reduction of 4-nitrophenol with NaBH_4_ for subsequent eight times as a model. As shown in Fig. 6, the catalytic efficiency of the first round reached nearly 99% and showed the value of 92% even after 8 times, demonstrating excellent stability and reutilization of present synthesized nanocomposites. The possible reason of catalytic degradation could be attributed to the following: first, the active sites of composites were covered by trace residual 4-NP or 4-AP. Second, there was a trace loss when the catalyst was recycled and washed. To avoid these situations, magnetic nanoparticles could be added to future designs to reduce loss. Figure 7 displayed the preparation of CNT-Cu_2_O nanocomposites and their catalytic properties on nitro-compounds. The scheme indicated that the preparation temperatures and time seemed to play an important role in regulating the sizes of Cu_2_O nanocrystal and next catalytic performance. l-ascorbic acid was added into the reaction system as a reducing agent and Cu_2_O were formed. And CNT acted as a substrate carrier to provide larger platform and anchoring sites to prevent agglomeration of Cu_2_O nanoparticles. In addition, the combination of CNT with Cu_2_O could enhance the process of electron transfer, which also increased intermolecular interactions between CNT and Cu_2_O. In other reported studies, the reduction reaction of 4-NP was catalyzed by Cu nanowires with a time of 13 min [23]. Thus, present synthesized CNT-Cu_2_O-1 achieved high catalytic performance, indicating potential and wide application in wastewater treatment and composite materials. The parameters with a reaction temperature of 30 °C with 1 h (CNT-Cu-30-1) could generate the small and uniform Cu_2_O nanocrystal in the formed composite material, while increased temperature (CNT-Cu-60-1) and extended preparation time (CNT-Cu-30-6) could produce large agglomerated blocks and obviously decrease the catalytic ability. It was obvious that the size and shape of Cu_2_O nanocrystal remarkably impacted on the catalytic activity. Therefore, present work provided potential exploration for the design and preparation of new nanocomposite materials for wide catalytic fields.

## Conclusions

In summary, we have presented a facile approach to synthesize CNT-Cu_2_O nanocomposites by taking advantage of a facile and low-cost method. Through in-depth analysis, the optimized synthesis condition for present CNT-Cu_2_O composite was at 30 °C for 1 h, which demonstrated Cu_2_O particles with a size of 30–50 nm and uniformly distributed on the surface of CNT. The optimized catalyst product was used for reducing 4-NP reaction to completely form 4-AP just within 35 s. Interestingly, the catalytic ability retained 92% after 8 cycles, which showed the well catalytic stability and potential application. The presence of CNT not only provided the function of template substrate, but also improved its mechanical properties and reusability stability. It is found that the synthesized products showed high performance on catalytic reduction of 4-NP even after eight subsequent repeated cycles. Current studies indicated that present synthesized CNT-Cu_2_O composite materials could be wide candidates for catalysts in the field of wastewater treatment and composite materials.

## Abbreviations

4-NP: P-nitrophenol
CNT: Carbon nanotube
CNT-Cu-30-1: Composite obtained under 30 °C and 1 h
CNT-Cu-30-6: Composite obtained under 30 °C and 6 h
CNT-Cu-60-1: Composite obtained under 60 °C and 1 h
SEM: Scanning electron microscope
TEM: Transmission electron microscope
XRD: X-ray diffraction
